# Modeling the growth curve in ducks: a sinusoidal model as an alternative to classical nonlinear models

**DOI:** 10.1016/j.psj.2024.103918

**Published:** 2024-05-31

**Authors:** Navid Ghavi Hossein-Zadeh

**Affiliations:** Department of Animal Science, Faculty of Agricultural Sciences, University of Guilan, Rasht, 41635-1314, Iran

**Keywords:** body weight, goodness of fit, mathematical function, model fitting, waterfowl

## Abstract

The present study aimed to apply a sinusoidal model to duck body weight records in order to introduce it to the field of poultry science. Using 8 traditional growth functions as a guide (Bridges, Janoschek, logistic, Gompertz, Von Bertalanffy, Richards, Schumacher, and Morgan), this study looked at how well the sinusoidal equation described the growth patterns of ducks. By evaluating statistical performance and examining model behavior during nonlinear regression curve fitting, models were compared. The data used in this study came from 3 published articles reporting 1) body weight records of Kuzi ducks aged 1 to 70 d, 2) body weight records for Polish Peking ducks aged 1 to 70 d, and 3) average body weight of Peking ducks aged 1 to 42 d belonging to 5 different breeds. The general goodness-of-fit of each model to the various data profiles was assessed using the adjusted coefficient of determination, root mean square error, Akaike's information criterion (**AIC**), and Bayesian information criterion. All of the models had adjusted coefficient of determination values that were generally high, indicating that the models generally fit the data well. Duck growth dynamics are accurately described by the chosen sinusoidal equation. The sinusoidal equation was found to be one of the best functions for describing the age-related changes in body weight in ducks when the growth functions were compared using the goodness-of-fit criteria. To date, no research has been conducted on the use of sinusoidal equations to describe duck growth development. To describe the growth curves for a variety of duck strains/lines, the sinusoidal function employed in this study serves as a suitable substitute for conventional growth functions.

## INTRODUCTION

Domestic ducks have a significant consumer base and are a valuable poultry in many parts of the world. The genera *Anas* and *Cairina* include domestic ducks. The majority of breeds are descended from the southern Chinese domesticated mallard duck (*Anas platyrhynchos*) (FAOSTAT, 2021). Recently, duck breeding has drawn a lot of attention from breeders looking to improve various economically important traits, like meat and egg production, by selecting accurate birds. Compared to other poultry species, ducks have several advantages, chief among them being their resistance to disease. They are resilient, good foragers, and simple to herd—especially in wetlands, where they have a propensity to congregate. According to data from the Food and Agriculture Organization of the United Nations (**FAO**), there are 1.17 million ducks in the world, and 32,35,471 tons of duck meat were produced in 2019 (FAOSTAT, 2021).

Growth, which is characterized as an increase in live weight or body dimension relative to age, is one of the most crucial characteristics of livestock. Growth curves provide a time-series explanation for changes in live weight or dimension (Ghavi Hossein-Zadeh and Golshani, 2016). Determining the optimal age for slaughter and suitable feeding practices can be achieved by analyzing an animal's growth performance over the course of its life. Growth curves have been the subject of more studies recently as a result of the availability of new models for testing and the development of new computational techniques for quicker and more accurate analyses (Souza et al., 2013). The growth curves of various animal classes have been suitably characterized by nonlinear mathematical functions that were empirically developed by plotting body weight against age.

Many growth models have been widely used in various species to explain how body weight develops. By combining data from several measurements into a small number of biologically meaningful parameters, these models help to simplify the phenomenon's interpretation and comprehension (Lambe et al., 2006; Ghavi Hossein-Zadeh, 2015). Growth curve analysis has several benefits in animal production: 1) it summarizes the most significant growth and developmental traits in 3 or 4 parameters; 2) it evaluates the treatment response profile over time; 3) it examines the relationship between the growth parameter and the animal's limit weight or asymptotic weight to identify the heaviest animals in a population at younger ages; and 4) it obtains the individual within and among variances, which are highly valuable in genetic assessments (Freitas, 2005). In order to modify the relationship between body weight and age through breeding and selection, growth curve parameters offer potentially helpful criteria. The ideal growth curve can be obtained by selecting for desired values of these parameters (Bathaei and Leroy, 1998). As breeding can increase early growth while limiting mature size and, consequently, maintenance requirements, it may be an appealing option for livestock producers to alter the shape of the growth curve (Lambe et al., 2006). Examining growth curves in ducks is essential because it offers pertinent data for developing strategic plans in the fields of nutrition and genetic breeding, including enhanced management, determining nutritional requirements, understanding the genetic variability of traits associated with growth, and evaluating the genetic potential of the animals for growth. These insights also aid in the decision-making process when deciding which technology to adopt (da Silva Marinho et al., 2013).

Because traditional growth models may not adequately capture the growth patterns of all poultry breeds or production systems, alternative equations are still required to estimate growth curves in poultry production. Alternative equations can better account for variables such as genetic variation, environmental conditions, and management techniques and provide greater flexibility in modeling growth curves. In general, alternative equations are still required for several reasons: 1) It is possible that different breeds of chickens have unique growth patterns that are not well represented by traditional models, 2) Growth rates and patterns can vary significantly depending on the production systems and management techniques used, and 3) Due to the impact that environmental factors such as temperature, humidity and feed quality can have on growth, differentiated modeling approaches are required. Previous studies have reported on the suitability of a sinusoidal function (as an alternative) to model the growth curve of turkeys, broilers, quails, and dairy heifers (Darmani Kuhi et al., 2018, 2019a,b,c). The production of ducks has developed into extremely specialized, well-coordinated systems. It is necessary to parameterize mathematical models of duck growth in order to optimize duck production systems, including assessing alternative management and nutritional strategies (Schinckel et al., 2013). Nevertheless, only a small number of studies have described the growth curves of various duck breeds, and the majority of earlier research used standard nonlinear models. As a result, research on duck growth curves are scarce in the literature, particularly when alternative models like sinusoidal models are used. By applying the sinusoidal function to duck growth data from the literature and contrasting it with 8 standard growth functions, viz. Janoschek, logistic, Morgan, Gompertz, von Bertalanffy, Richards, Schumacher, and Bridges, the current study aimed to introduce a sinusoidal function in research on the weight development of ducks.

## MATERIALS AND METHODS

### Data Sources

Body weight records for Kuzi ducks (Padhi et al., 2022), Polish Pekin ducks (Kokoszyński et al., 2019), and Peking duck breeds (Wild et al., 2019) were used in this study. Data from Padhi et al. (2022) presented body weights at 1, 7, 14, 21, 28, 35, 42, 49, 56, and 70 d of age in the S2 generation of Kuzi ducks of India selected for higher body weight at 8 wk in generations S0 and S1. Data presented by Kokoszyński et al. (2019) determined body weights at 1, 7, 14, 21, 28, 35, 42, and 49 d of age in the P33 line of Pekin ducks of Polish origin included in the Genetic Resources Conservation Program in Poland. Furthermore, the information provided by Wild et al. (2019) were the average body weights of 2380 Peking duck breeds, ranging in age from day-old to 42 d, belonging to 5 different breeds: Cherry Valley, Maple Leaf, Wichmann, Grimaud Frères, and Orvira (Supplementary Table 1).

### Nonlinear Models

To model the relationship between body weight and age, the following models were fitted to the data: Bridges, Janoschek, logistic, Gompertz, Schumacher, Richards, sinusoidal, von Bertalanffy, and Morgan (Table 1).

**Table 1:** Functional forms of nonlinear models for describing the growth curve of ducks.

Model	Functional form
Bridges	$y=W_{0}+\left(1-e^{-kt^{m}}\right)$
Janoschek	$y=a-(\left(a-W_{0}\right)e^{-kt^{m}})$
Logistic	$y=\frac{a}{1+be^{-kt}}$
Gompertz	$y=ae^{-be^{-kt}}$
Von Bertalanffy	$y=a{\left(1-be^{-kt}\right)}^{3}$
Richards	$y=\frac{a}{{\left(1-be^{-kt}\right)}^{\frac{1}{m}}}$
Schumacher	$y=\frac{ab^{2}k}{{\left(t+b\right)}^{2}}e^{\left(\frac{bkt}{t+b}\right)}$
Morgan	$y=ab^{k}k\frac{t^{k-1}}{{\left(t^{k}+b^{k}\right)}^{2}}$
Sinusuidal	$y=y_{0}+a\times \sin\left(\frac{2\pi t}{b}+c\right)$

y= represents body weight at age t (day); a= represents asymptotic weight, which is interpreted as mature weight; and b= is an integration constant related to initial animal weight. The value of b is defined by the initial values for y and t; k= is the maturation rate, which is interpreted as weight change in relation to mature weight to indicate how fast the animal approaches adult weight; m= is the parameter that gives shape to the curve by indicating where the inflection point occurs; W_0_= is initial body weight. For sinusoidal function, *a* is the amplitude, $y_{o}$ is the vertical offset and *c* is the phase shift. This sinusoidal function is periodic with period *b*. Also, for sinusoidal function, W_0_=y_0_+a×sin(c), and final weight is calculated by a+ y_0_. For Schumacher and Morgan models, the asymptotic weight is not an estimated parameter.

### Statistical Analysis

Using the NLIN and MODEL procedures in SAS (SAS Institute, 2002), 8 conventional nonlinear models were fitted independently to duck body weight records, and the parameters were estimated. The NLIN process yields estimates of a nonlinear model's parameters using least squares or weighted least squares. It is necessary to specify the model (using a single dependent variable) as well as the names and initial values of the parameters to be estimated for each nonlinear model to be examined (SAS Institute, 2002). The Gauss-Newton method was employed as the iteration technique when fitting non-linear functions. The NLIN procedure first assesses the parameters' starting value specifications before initializing this process. The NLIN procedure determines the set of parameter values resulting in the lowest residual sum of squares (**RSS**) if a grid of values is specified by evaluating the RSS at each combination of parameter values. For the first iteration step, these parameter values are employed (SAS Institute, 2002). Models where a system of one or more nonlinear equations is comprised of the relationships between variables are analyzed using the MODEL procedure. Nonlinear simultaneous equation model forecasting, simulation, and estimation are the main applications of the MODEL procedure (Ghavi Hossein-Zadeh, 2014). Using the nonlinear regression method of SigmaPlot 15.0 (Systat Software, Inc., San Jose, CA), the sinusoidal function was fitted to the ducks' growth curve. Model parameter estimation was done iteratively using the Marquardt-Levenberg algorithm. The iterative process required the initial values of the parameters to be provided. The initial values adopted had no bearing on the final estimates.

Using the adjusted coefficient of determination ($R_{adj}^{2}$), residual standard deviation or root means square error (**RMSE**), Durbin-Watson (**DW**) statistic, Akaike's information criterion (**AIC**), and Bayesian information criterion (**BIC**), the models were evaluated for goodness of fit, or quality of prediction.

The following formula was used to calculate $R_{adj}^{2}$:

$$R_{adj}^{2}=1-\left[\frac{\left(n-1\right)}{\left(n-p\right)}\right]\left(1-R^{2}\right)$$

Where, the multiple coefficient of determination is denoted by $R^{2}$($R^{2}=1-\frac{RSS}{TSS}$), n denotes the number of observations (data points), p denotes the number of parameters, RSS stands for residual sum of squares, and TSS stands for total sum of squares. The $R^{2}$value is used to determine how much of the total variation around the mean of the trait can be attributed to the growth curve model. The $R^{2}$always falls within the range of 0 to 1, with the model considered satisfactory when $R^{2}$ is close to 1.

RMSE is a form of standard deviation that is calculated in the following way:

$$RMSE=\frac{RSS}{\sqrt{n-p-1}}$$

The RSS represents the error in the data, with n being the number of observations and p being the number of parameters in the equation. The RMSE value is a critical factor in evaluating the appropriateness of growth curve models, with the most favorable model being the one with the lowest RMSE.

The residuals from the regression analysis were examined for the presence of autocorrelation using the DW statistic. It is possible that the function is not suitable for the data, as indicated by the existence of autocorrelated residuals. The values of the DW statistic span from 0 to 4. When the value is close to 2, autocorrelation is absent; when the value is close to 0, autocorrelation is positive; and when the value is close to 4, autocorrelation is negative (Ghavi Hossein-Zadeh, 2014). The following formula was used to determine DW:

$$DW=\frac{\sum_{t}^{n}{\left(e_{t}-e_{t-1}\right)}^{2}}{\sum_{t=1}^{n}e_{t}^{2}}$$

Where, $e_{t}$denotes the residual at time e, and $e_{t-1}$ presents residual at time t-1.

Using the following equation (Burnham and Anderson, 2002), AIC was calculated:

AIC=n×ln(RSS)+2p

Because AIC modifies the RSS according to the model's parameter count, it is a useful statistic for comparing models of varying complexity. In model comparison, a lower AIC numerical value denotes a better fit.

By penalizing the (log) maximum likelihood with a term associated with model complexity, BIC combines maximum likelihood (data fitting) and model selection:

$$BIC=n\ln\left(\frac{RSS}{n}\right)+p\ln\left(n\right)$$

When comparing models, a lower BIC number denotes a better fit.

Following the selection of the best-fitting function, the first derivative of the function with respect to time was used to compute the absolute growth rate, or AGR ($\frac{\partial y}{\partial t}$). The average growth rate of animals in a population is actually represented by the AGR, which actually represents weight gain per unit of time. In this case, this represents the estimated daily weight gain over the course of a growing period (Ghavi Hossein-Zadeh, 2015).

## RESULTS

There existed magnitude differences between the various functions for the final or asymptotic body weights of different duck breeds (Table 2). For most functions, the asymptotic weight estimate represented by the growth model parameter “a” is the mature weight of the duck. The asymptotic weights of the Kuzi, Polish Pekin, and Peking duck breeds predicted using the sinusoidal function as the best-fitting model were 1,534.98, 2,030.05 and 4,519.57 g, respectively. The asymptotic body weights predicted by other models were generally higher than these values. Compared to Kuzi and Peking duck breeds, Polish Pekin ducks had higher values for the growth model parameter “k” (maturation rate) for most functions (Table 2). This suggested that Polish Pekin ducks reached adult weight earlier compared to the other 2 breeds.

**Table 2:** Parameter estimates for the different growth models in ducks.

Breed	Parameter	Model								
		Bridges	Janoschek	Logistic	Gompertz	Von Bertalanffy	Richards	Schumacher	Morgan	Sinusuidal
Kuzi, Padhi et al. (2022)	y_0_	-	-	-	-	-	-	-	-	762.8032
	W_0_	50.3691	50.3691	-	-	-	-	-	-	-
	a	1,572.4030	1,622.7710	1,568.0100	1,718.5880	1,840.0630	1,666.3600	46.2627	215,425.0000	772.1740
	b	-	-	18.5560	4.0519	0.8455	-1.2669	24.1314	102.7760	150.8056
	c	-	-	-	-	-	-	-	-	5.0027
	k	0.0008	0.0008	0.0926	0.0533	0.0402	0.0622	0.3990	2.5069	-
	m	1.9403	1.9403	-	-	-	0.2246	-	-	-
Polish Pekin, Kokoszyński et al. (2019)	y_0_	-	-	-	-	-	-	-	-	1,031.9913
	W_0_	66.2416	66.2431	-	-	-	-	-	-	-
	a	2,226.5940	2,292.8320	2,110.7400	2,485.1810	2,869.9300	2,371.4450	69.5656	245,558.7000	998.0614
	b	-	-	20.7380	4.0690	0.8204	-0.9274	22.5424	85.8948	115.5238
	c	-	-	-	-	-	-	-	-	4.8717
	k	0.0010	0.0010	0.1144	0.0595	0.0406	0.0692	0.4229	2.5469	-
	m	1.9495	1.9495	-	-	-	0.1737	-	-	-
Peking breeds, Wild et al. (2019)	y_0_	-	-	-	-	-	-	-	-	2,266.2669
	W_0_	51.8410	51.8416	-	-	-	-	-	-	-
	a	6,045.7650	6,097.6090	4,183.0000	5,647.2660	7,691.3450	6,899.5060	120.9637	651,940.5000	2,253.2977
	b	-	-	27.3406	4.4248	0.8460	0.7187	24.9350	97.6016	126.6430
	c	-	-	-	-	-	-	-	-	4.8444
	k	0.0010	0.0010	0.1210	0.0546	0.0319	0.0380	0.4018	2.6174	-
	m	1.8233	1.8233	-	-	-	-0.2454	-	-	-

**Figure 1:**
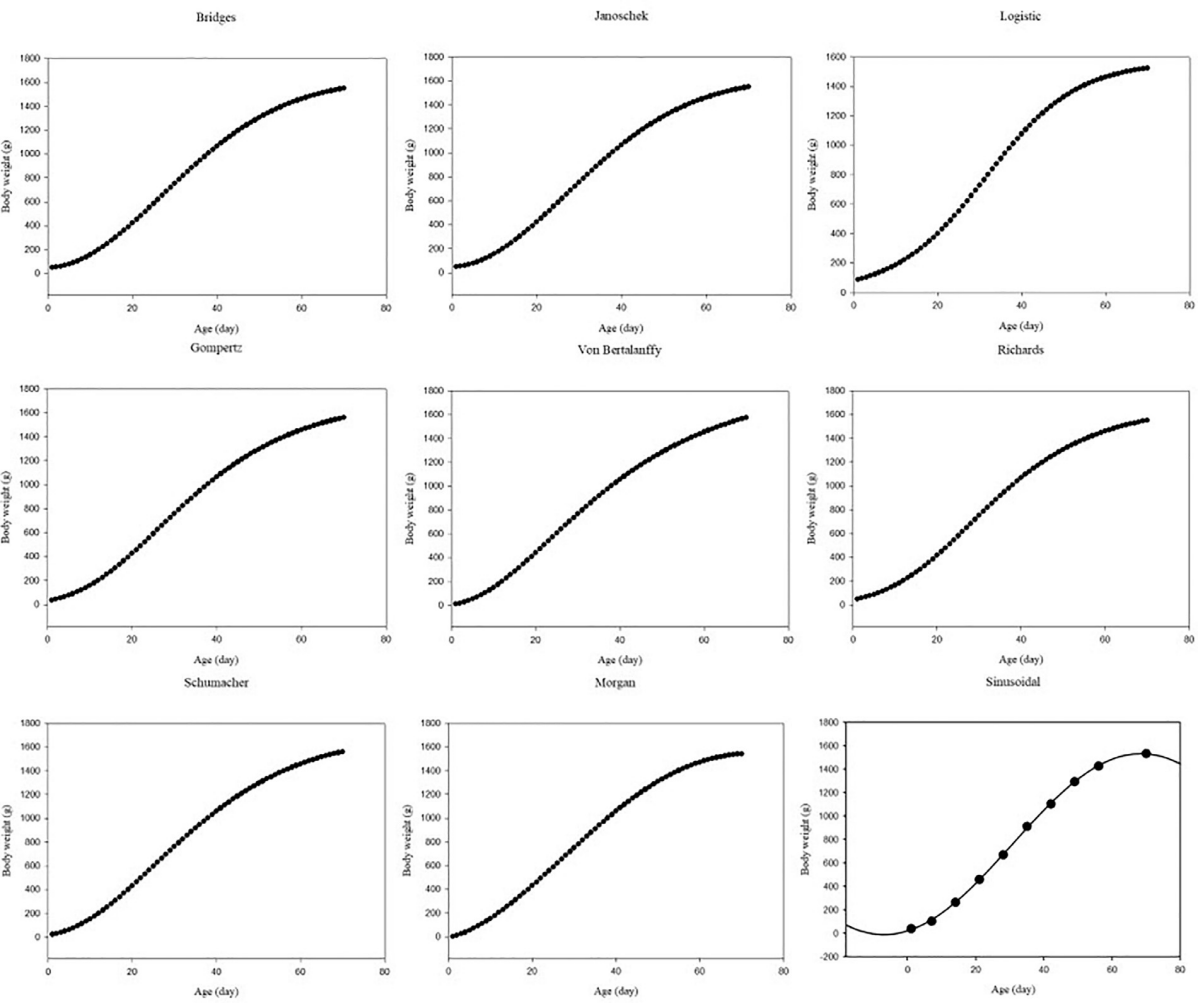
Predicted body weights as a function of age, determined using different growth models for Kuzi ducks.

Observed body weights of Kuzi, Polish Pekin, and Peking duck breeds were reported in Kokoszyński et al. (2019), and Wild et al. (2019), Padhi et al. (2022), respectively. As previously mentioned, there was a tendency for body weight to increase as age increased. Figure 1 shows the predicted body weights for Kuzi ducks based on different growth models as a function of age. According to the growth curves, there were generally sigmoidal increasing trends with age. For the data sources, the logistic model indicated an initial body weight that was overestimated. The values for the Gompertz, Schumacher, Janoschek, Bridges, and Richards were generally quite similar to the initial body weights. However, initial body weight for the data sources was often underestimated by the Von Bertalanffy, Morgan, and sinusoidal models. Supplementary Figures 1 and 2 show the predicted body weights as a function of age for the Peking duck breeds and Polish Pekin ducks, respectively, based on different growth models.

Although both the sinusoidal and Gompertz models had the highest $R_{adj}^{2}$ value for Polish Pekin, there were minimal differences in $R_{adj}^{2}$ values between the models for Kuzi, Polish Pekin, and Peking duck breeds. For Kuzi and Peking duck breeds, however, the sinusoidal function yielded the highest $R_{adj}^{2}$ value. However, the logistic model produced the smallest values of $R_{adj}^{2}$ for all duck breeds in this study (Table 3). DW values ranged from 0.8467 (for logistic) to 2.9006 (for sinusoidal) in Kuzi ducks, from 1.6013 (for logistic) to 3.4370 (for Richards) in Polish Pekin, and from 1.1155 (for logistic) to 2.6607 (for sinusoidal) in Peking duck breeds. The majority of the growth models in this study had DW values that were, on average, moderate and varied around 2. The logistic model had the highest RMSE values, while the sinusoidal function produced the lowest RMSE values for Kuzi, Polish Pekin, and Peking duck breeds. Additionally, for the Kuzi and Peking duck breeds, sinusoidal function had the lowest AIC and BIC values, and for the Polish Pekin, sinusoidal and Gompertz models had the lowest values. However, for the Kuzi, Polish Pekin, and Peking duck breeds, the logistic model had the highest AIC and BIC values (Table 3). As a result, the sinusoidal model was determined to be the most effective function for fitting the growth curve of the Peking, Polish, and Kuzi duck breeds. However, among all duck breeds, the logistic model had the worst fit to the growth curve.

**Table 3:** Comparison of goodness of fit for different growth curves in ducks.

Breed	Statistics	Model								
		Bridges	Janoschek	Logistic	Gompertz	Von Bertalanffy	Richards	Schumacher	Morgan	Sinusuidal
	$R_{adj}^{2}$	0.9991	0.9991	0.9970	0.9988	0.9970	0.9990	0.9985	0.9990	0.9997
	DW	1.9527	1.9527	0.8467	1.9321	1.3511	2.0184	1.5839	0.9077	2.9006
Kuzi, Padhi et al. (2022)	RMSE	16.5897	16.5897	30.4244	18.9329	30.3482	17.7253	21.5065	17.6102	9.2164
	AIC	82.0932	82.0932	93.7640	84.2772	93.7139	83.4174	86.8262	82.8285	70.3372
	BIC	60.2777	60.2777	71.6459	62.1591	71.5958	61.6019	64.7081	60.7104	48.5217
	$R_{adj}^{2}$	0.9986	0.9986	0.9970	0.9990	0.9977	0.9989	0.9985	0.9978	0.9990
	DW	3.3071	3.3071	1.6013	3.2858	2.2428	3.4370	2.7143	1.7400	3.4166
Polish Pekin, Kokoszyński et al. (2019)	RMSE	27.7946	27.7946	40.2314	23.7455	35.6220	24.6361	28.6788	34.7150	23.3750
	AIC	72.2879	72.2879	77.9898	69.5540	76.0429	70.3579	72.5741	75.6302	69.5170
	BIC	55.9702	55.9702	61.5926	53.1567	59.6457	54.0402	56.1769	59.2330	53.1992
	$R_{adj}^{2}$	0.9997	0.9997	0.9969	0.9996	0.9997	0.9997	0.9997	0.9996	0.9998
	DW	2.2583	2.2583	1.1155	1.9332	2.2068	2.4028	2.3543	1.4225	2.6607
Peking breeds, Wild et al. (2019)	RMSE	25.3281	25.3281	79.2005	29.8841	24.1347	25.6328	23.8667	28.3278	20.1142
	AIC	70.8011	70.8011	88.8272	73.2327	69.8139	70.9924	69.6353	72.3770	67.1132
	BIC	54.4834	54.4834	72.4300	56.8355	53.4167	54.6747	53.2381	55.9798	50.7954

$R_{adj}^{2}$: Adjusted coefficient of determination; RMSE: Root means square error; DW: Durbin–Watson; AIC: Akaike information criteria; BIC: Bayesian Information Criteria.

**Figure 2:**
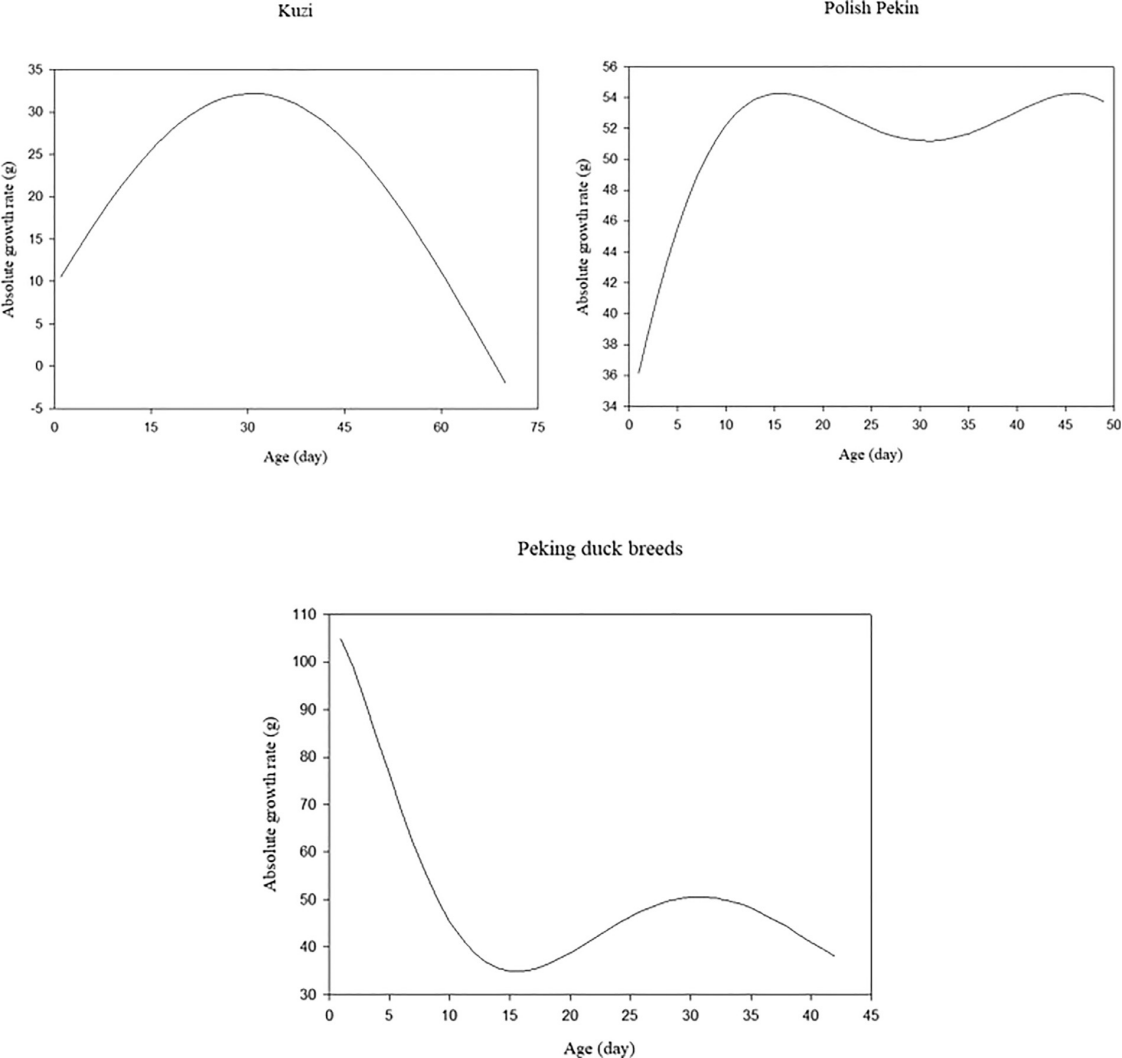
Absolute growth rate (**AGR**) for different duck breeds based on sinusoidal model.

Figure 2 displays the AGR values for various duck breeds as a function of time, based on the first derivative of the sinusoidal model. In Kuzi ducks, AGR values progressively rose with increasing age until d 31 and then declined. The AGR values of Polish Pekin ducks showed a slight increase over time up to 16 d of age. Afterwards, they showed a slight irregular variation with an almost linear trend. AGR values ​​for Peking duck breeds decreased steadily with age, reaching a plateau at 16 days of age before showing a slight, irregular change with a predominantly linear pattern.

## DISCUSSION

While chickens produce the majority of poultry meat and eggs, ducks are used in some regions of the world to produce sizable amounts of meat. Waterfowl are marketed at relatively low prices and provide high-quality nutrition in their meat and eggs. The production of ducks and geese makes up roughly 7.5% of the total amount of poultry meat produced worldwide (Stipkovits and Szathmary, 2012). Growth rate, feed intake, and feed conversion rate are among the fundamental biological data that must be dependable when planning and calculating duck production facilities. Assessing the environmental effects of duck production also requires consideration of these data (Wild et al., 2019).

Growth curves are helpful tools for showing how body weight changes over the course of growth and for providing relevant and useful data for feeding and breeding programs (Maruyama et al., 1998; Aggrey, 2004). Given their ability to predict future growth at any age, growth curves may be utilized for animal pre-selection (Tekel et al., 2005; Darmani Kuhi et al., 2019b). Growth curves have been described by a variety of mathematical functions. Additionally, precise estimation of daily weight gain through the use of growth functions will facilitate more accurate determination of the bird's energy and nutrient requirements, thereby facilitating the development of more functional diets. Following the selection of a suitable growth curve model, an individual animal's growth curve phase can be chosen directly. It is crucial to devise the best plan of action for modifying the growth model's parameters in order to achieve the intended growth shape (Ghavi Hossein-Zadeh, 2014,^2015^). To the best of the author's knowledge, not much is known about the application of trigonometric functions (like the suggested sinusoidal functions) (Darmani Kuhi et al., 2018) to explain how poultry growth has changed over time. The ability of a sinusoidal equation to characterize the relationship between body weight and age in ducks was assessed in this study. The fitting results obtained from the sinusoidal model were compared to 8 common growth functions, namely logistic, Von Bertalanffy, Gompertz, Janoschek, Richards, Schumacher, Morgan, and Bridges. Many distinct curve shapes can be described by the sinusoidal equation. All functions provided a good fit to the data profiles, according to a comparison of the models based on their behavior and statistical performance. Overall, the statistical criteria used for evaluating the growth models revealed some significant variations in the functions that represent the future growth of ducks. The estimated goodness of fit criteria showed that the sinusoidal growth function was found to be the most suitable for explaining the age-related changes in bodyweight in ducks when growth functions were compared, although the suitability of the models may depend on the data and gender (Darmani Kuhi et al., 2019a). However, choosing the optimal function necessitates paying close attention to how animals grow in various environmental settings (Dogan et al., 2010). This is particularly important when a specific data set is obtained under previously undefined specific conditions (for a specific bird species, for a specific feeding regime, or when ducks are kept in a specific climate or environment) (Darmani-Kuhi et al., 2019b).

Direct comparisons between models were most facilitated by the asymptotic weight parameter “a” which represents an estimate of the mature weight. The maximum growth response for birds was represented by the asymptotic weight parameter, and the models used in this study had different estimates of this parameter. Both environmental and genetic factors had a direct influence on asymptotic weight (Ogunshola et al., 2020). Another important factor to consider is the growth model parameter “k,” which is defined as the rate of maturation. This parameter indicates the rate at which a bird will grow in order to reach its mature or asymptotic weight. The Peking ducks had higher values of this parameter compared to two other breeds, which were estimated by most models. Birds with higher “k” values are likely to reach mature or asymptotic weight earlier than birds with lower values of this parameter, because high “k” values indicate advanced maturity (Ghavi Hossein-Zadeh, 2014).

Consistent with the results of this study, other researchers reported high coefficients of determination when fitting nonlinear models to describe the growth curve in ducks (Faridi et al., 2014; Asiamah et al., 2020; Lan et al., 2020). High values of the coefficients of determination or values close to one indicate better fit when comparing the models. It was determined that there were both positive and negative autocorrelations between the residuals for some models based on the values of DW obtained from fitting the nonlinear models of growth curve in the current study. In addition, some models provided residuals without autocorrelations. The remaining autocorrelation appears to have caused little or no problems in this study. A positive error for one observation raises the likelihood of a positive error for another observation, a phenomenon known as positive autocorrelation or serial correlation (Ghavi Hossein-Zadeh, 2014).

Several studies mainly compared different traditional models to describe the growth curve in ducks. Almeida et al. (2018) compared the growth curves of native duck lineages (Catolé and Paysandu ducks) using 5 nonlinear models (Brody, Richards, Gompertz, Von Bertalanffy, and logistic). The growth curve fitted by the logistic model was the best fit, and the AGR for the Catolé duck indicated that females reached their maximum AGR at about 30 d. Anang et al. (2017) used 6 different models of Brody, Gompertz, logistic, Morgan Mercer Flodine, Richards, and Von Bertalanffy to analyze the growth curve of Rambon ducks. The results showed that while all of the growth models were a good fit for both male and female ducks, the logistic model offered the best growth curve fit. Asiamah et al. (2020) utilized the logistic, Gompertz, and Von Bertalanffy models to determine the growth pattern of male and female Leizhou Black Ducks. They determined that the logistic, Gompertz, and Von Bertalanffy models had comparable accuracy rates, with the Von Bertalanffy model being identified as the top-performing model. Faridi et al. (2014) presented two alternative growth models of the Lomolino and extreme value function for predicting body, carcass, and breast weight in ducks. After comparing two functions' abilities to standard growth functions of Gompertz, exponential, Richards, and generalized Michaelis-Menten, they came to the conclusion that the alternative functions are more desirable than the standard functions because they are flexible and require fewer parameters. Kaewtapee et al. (2011) examined the growth models used in nonlinear regression analysis (asymptotic exponential, logistic, cubic curvilinear, and Gompertz) and artificial neural networks (the multilayer perceptron method (**MLP**) and the radial basis function (**RBF**)) in Cherry Valley ducks. They came to the conclusion that, when it came to estimating the body weight of Cherry Valley ducks, RBF outperformed MLP in prediction accuracy, but not Gompertz and cubic curvilinear functions. Lan et al. (2020) compared 4 sigmoidal models (Richards, Gompetz, logistic, and Morgan-Mercer-Flodin) to describe the growth curves of local Cihateup ducks in West Java, with the Richards function emerging as the most suitable model based on high R^2^ values and low standard errors. Önk et al. (2018) determined the growth models of male and female ducks using the Gompertz model and 3-parameter logistic regression. The accuracy rate of the Gompertz and 3-parameter logistic models for estimating duck growth was comparable. Because the Gompertz model has fewer iterations than the 3-parameter logistic model, they determined that it is suitable for the data structure of their study. Thinh et al. (2021) modeled the growth curve of Eastern spot-billed ducks raised in Vietnam using 6 growth models: Gompertz, Brody, logistic, Richards, Bridges, and Janoschek. Their study demonstrated the viability of modeling Eastern spot-billed duck growth using the Gompertz growth model. The differences in time units, using months rather than days or weeks, the use of different growth models, genetic variance among breeds, the use of feeds with different formulas, and different breeding environments may account for discrepancies in findings across various studies (Goliomytis et al., 2006; Thinh et al., 2021).

Variations in the growth curve properties of ducks may account for the notable discrepancies in the goodness of fit of the various breeds' functions. The differences in body weight records, animal breeds, number of data points, and mathematical form of the models could all have contributed to the variation in model fit across studies. Genetic and environmental factors both contribute to variations in growth curves. The asymmetry surrounding the inflection point may have contributed to the logistic model's poor performance in this study; in contrast, the sinusoidal model offered the best fit to the growth curve. The functions' flexibility in adapting different types of data and the asymmetry surrounding the inflection point were the reasons behind the sinusoidal model's advantage (Darmani Kuhi et al., 2019a). The ability to capture periodic growth patterns, simplicity and ease of interpretation are other key advantages of modeling livestock growth curves with a sinusoidal function. Compared to simpler models such as linear or exponential models, the sinusoidal model supports more complex and adaptive growth patterns. Seasonal fluctuations in growth rates can also be easily accounted for by adjusting the sinusoidal functions. Because the sinusoidal model mimics cyclic patterns commonly observed in animal growth processes, it is of biological significance. For example, growth rates may fluctuate throughout the year due to factors such as food availability or changes in the environment. Nonlinear growth patterns that may not be explained by linear or exponential models can be accounted for by the sinusoidal model. This can be particularly helpful for animals that are growing rapidly at certain stages of development. The sinusoidal model, which often fits growth data better than other models, can be used to more accurately predict animal growth patterns. Nevertheless, modeling the growth curves of livestock with a sinusoidal function has certain disadvantages. One of these drawbacks is that real data might not always show the assumed smooth and continuous growth pattern. It is possible that sinusoidal functions have difficulty accurately capturing abrupt shifts or fluctuations in growth rates. Additionally, fitting complex growth patterns when using a sinusoidal function can be challenging and there is a possibility of data overfitting.

The disparities in growth, maturation, and relative growth rates between functions are indicative of existing discrepancies in the functions' propensity to fit the data. Given their ability to change in response to selection, growth curves are essential for comprehending and developing breeding programs. To assess an animal's genetic potential for growth, nonlinear functions have been widely used to represent size changes with age (Ozoje et al., 2015).

Given their correlation with other traits and production efficiency, early estimation of mature weight and growth rate in relation to body size may be significant for selection purposes (Darmani Kuhi et al., 2019c). The economics of production are closely linked to the rates of maturing, growth, and maturity size. As a result, livestock breeders have focused on these crucial characteristics. Economic returns could be positively enhanced by utilizing these parameters in growth models through curve fitting with live-weight-age data (Salako, 2014).

The AGR values show how much an animal's growth is emphasized in relation to its lifespan. As a result, AGR stands for average weight gain of the animals, calculated along the growth trajectory and obtained per unit of time. Therefore, AGR records provide a more comprehensive and dynamic insight into poultry growth patterns than relying solely on body weight records. This information enables better knowledge of growth trajectories and patterns, such as periods of rapid growth, growth plateaus or possible growth anomalies (Boonkum et al., 2024). Early detection of differences in growth performance can be facilitated by AGR values. Keeping track of weight gain trends allows for early identification of any irregularities in growth patterns, which in turn facilitates timely interventions or modifications in management techniques. This can involve adjusting feeding schedules, addressing health concerns, or implementing specific strategies to enhance growth potential (Boonkum et al., 2024). It is important to investigate this trait by giving ducks unrestricted access to food and by avoiding demanding schedules while they are growing more quickly. The results for the AGR of Polish Pekin and Kuzi ducks show that the ducks reached their maximum growth rate at earlier ages, when their growth speed was at its highest. By the end of the analysis period, Kuzi ducks' AGR was very small, indicating that they had not gained much weight at all. However, for Peking duck breeds, the AGR gradually dropped during the earlier weeks when the ducks had attained their slowest growth rate. The maximum AGR is reached at an inflection point, after which the growth rate steadily declines, which explains the declining trend. Another possible reason for the decline in AGR could be inadequate management. Therefore, it is important to make feed management improvements to enhance the animals' weight gain, particularly during this phase (Pires et al., 2017).

## CONCLUSIONS

The outcome of a research project can be greatly enhanced by choosing a flexible and accurate model to predict the growth curve, and the model's accuracy guarantees its use. When compared to other conventional growth functions, the recently introduced sinusoidal function performed reasonably well in explaining the relationships between weight and age in ducks. Two desirable features of this function are the ability to capture periodic growth patterns and its flexibility. Additionally, it fit the investigated data with ease. The results of this study showed that the sinusoidal function introduced here could be regarded as a suitable substitute for standard growth functions when modeling growth patterns in ducks, based on various criteria to measure goodness of fit statistics. This is a key advantage of the recently proposed sinusoidal model. However, further research into the use of the introduced function is desirable as this is the first study to use it to predict duck growth patterns.

## DISCLOSURES

The authors declare no conflicts of interest.

## Appendix Supplementary materials

Supplementary Figure 1. Predicted body weights as a function of age, determined using different growth models for Polish Pekin ducks Image, image 1

Supplementary Figure 2. Predicted body weights as a function of age, determined using different growth models for Peking duck breeds Image, image 2

Image, application 3

## Footnotes

Supplementary material associated with this article can be found in the online version at doi:10.1016/j.psj.2024.103918.
