# Supplementary figures and images for: Modeling the growth curve in ducks: a sinusoidal model as an alternative to classical nonlinear models
Source: Poult Sci. 2024 May 31;103(8):103918. doi: 10.1016/j.psj.2024.103918 (PMC11250861; doi:10.1016/j.psj.2024.103918)

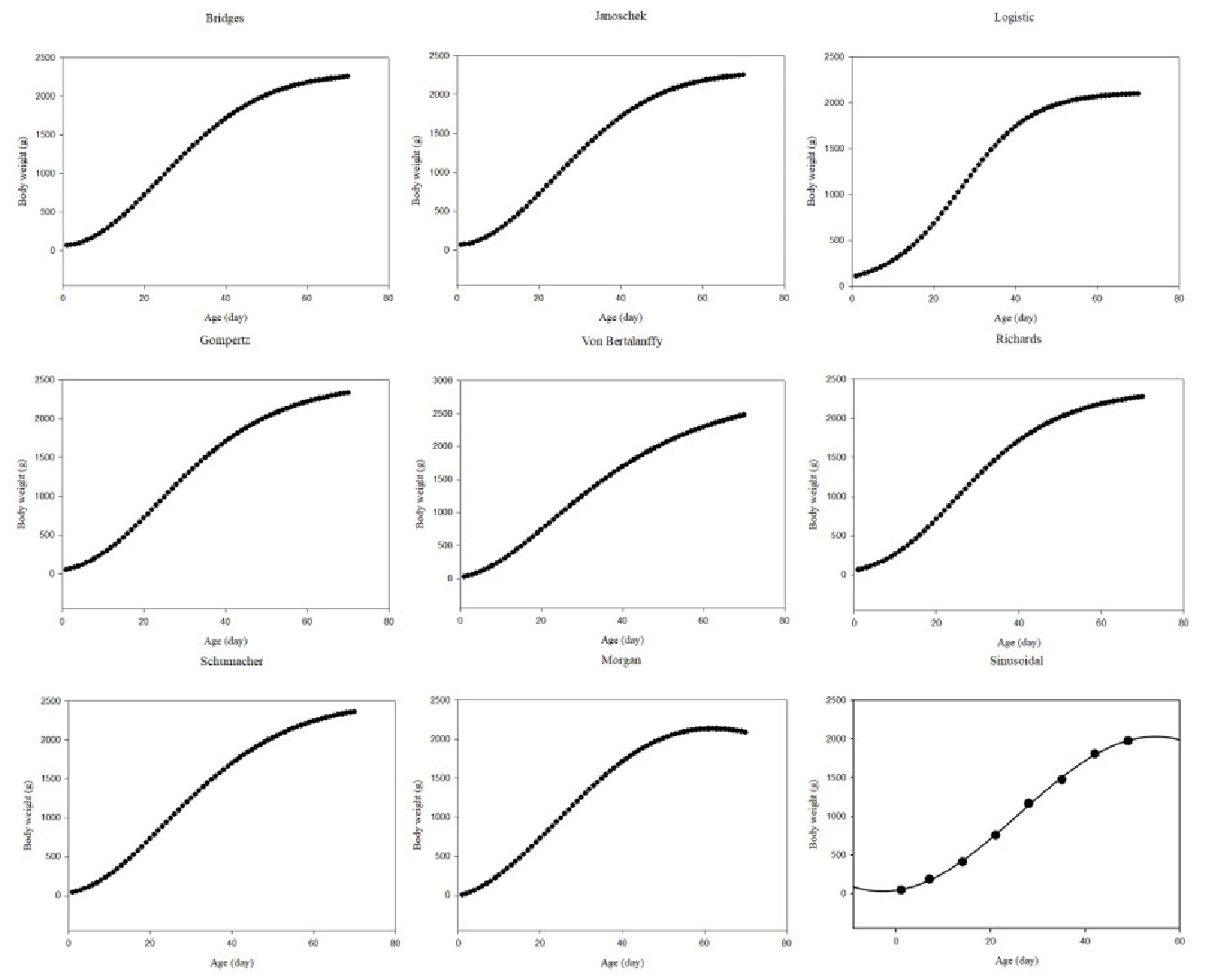

Supplement: Supplementary file 1 — Supplementary Figure 1. Predicted body weights as a function of age, determined using different growth models for Polish Pekin ducks [file mmc1.jpg]

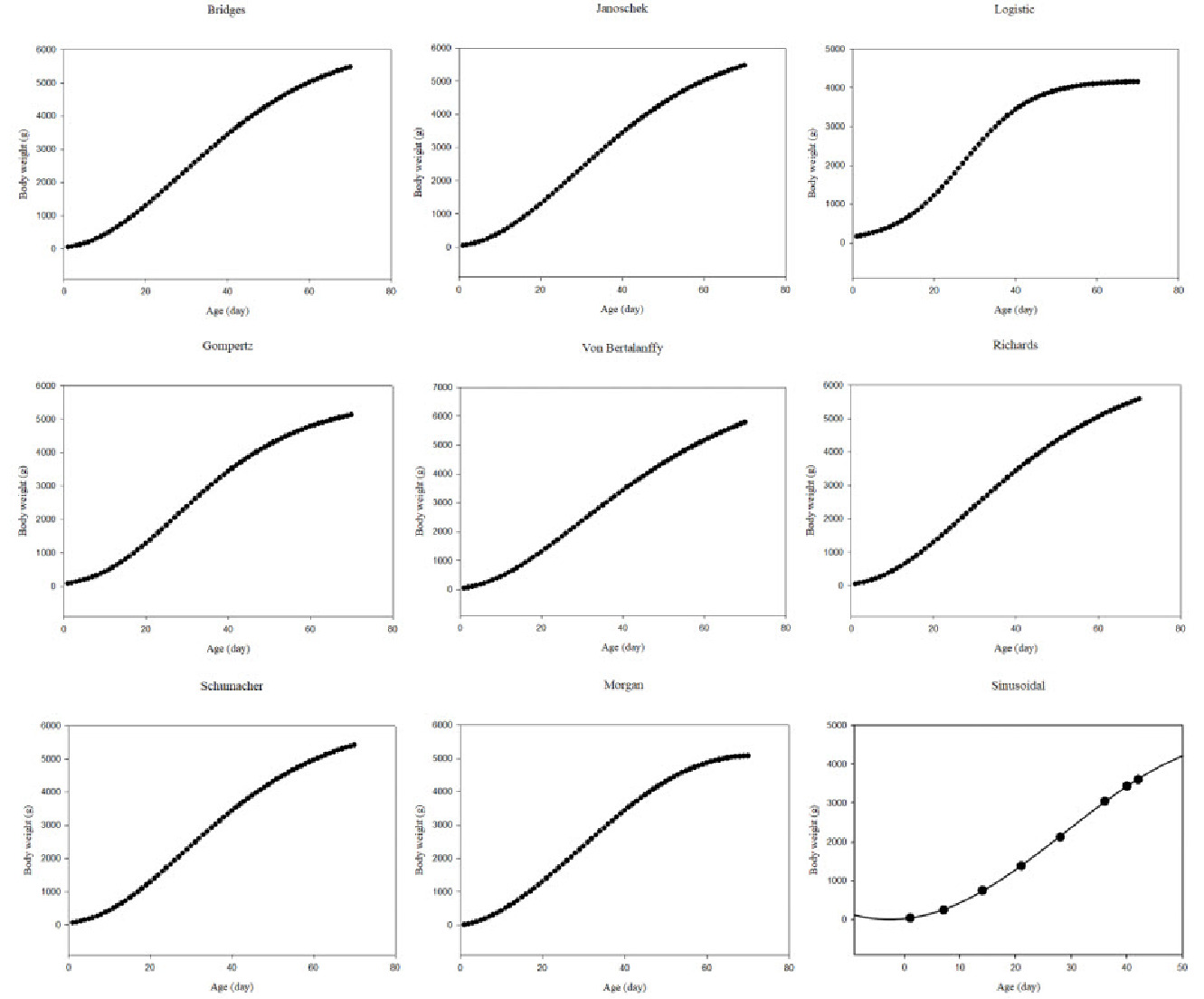

Supplement: Supplementary file 2 — Supplementary Figure 2. Predicted body weights as a function of age, determined using different growth models for Peking duck breeds [file mmc2.jpg]
