# Supplementary material for: Modeling the growth curve in ducks: a sinusoidal model as an alternative to classical nonlinear models
Source: Poult Sci. 2024 May 31;103(8):103918. doi: 10.1016/j.psj.2024.103918 (PMC11250861; doi:10.1016/j.psj.2024.103918)
Supplement: Supplementary file 3 [file mmc3.docx]

Supplementary Table 1. Data sets for modeling growth curves in ducks

| Kuzi duck^*^ | |  | Polish Pekin duck^**^ | |  | Peking duck breeds^***^ | |
| --- | --- | --- | --- | --- | --- | --- | --- |
| Age (day) | Body weight (g) |  | Age (day) | Body weight (g) |  | Age (day) | Body weight (g) |
| 1 | 41.12 |  | 1 | 51.3 |  | 1 | 51.4 |
| 7 | 105.6 |  | 7 | 191 |  | 7 | 257 |
| 14 | 266.2 |  | 14 | 415 |  | 14 | 758 |
| 21 | 461.49 |  | 21 | 759 |  | 21 | 1393 |
| 28 | 671.13 |  | 28 | 1170 |  | 28 | 2132 |
| 35 | 913.29 |  | 35 | 1476 |  | 36 | 3055 |
| 42 | 1105.63 |  | 42 | 1811 |  | 40 | 3442 |
| 49 | 1295.65 |  | 49 | 1977 |  | 42 | 3608 |
| 56 | 1428.84 |  |  | |  |  | |
| 70 | 1535.84 |  |  |  |  |  |  |

^*^ Padhi et al. (2022)

^**^ Kokoszyński et al. (2019)

^***^ Wild et al. (2019)
